# INITIAL EFFICACY OF AN APP-BASED MINDFULNESS MEDITATION FOR BEREAVED KOREAN OLDER ADULTS DURING THE PANDEMIC

**DOI:** 10.1093/geroni/igac059.1227

**Published:** 2022-12-20

**Authors:** Soonhyung Kwon, Jaesung An

**Affiliations:** University of Illinois Urbana Champaign, Urbana Champaign, Illinois, United States; California State University East Bay, Cupertino, California, United States

## Abstract

Given the onset of COVID-19, older adults who recently lost their significant others feel more stressed. There yet exist a study utilizing smartphones for web-based delivery of mindfulness intervention among bereaved older adults. Therefore, this study aimed to test the initial efficacy of an app-based mindfulness-meditation (AMM) to alleviate stress and depressive symptoms and improve stress resistance, social support, and self-esteem in Korean older adults experiencing bereavement. Participants included 22 Korean older adults who had been bereaved within the preceding year. AMM involved sound therapy, breathing exercises, and narrated meditation sessions, and the program was conducted over eight weeks. The linear regression results showed that stress level among participants was significantly lower after the intervention, with decreased scores from the baseline. By confirming that AMM is an effective way of reducing stress, more active usage of devices like smartphones should be promoted to develop mental health interventions for older adults.

